# Initial intramuscular dissection as a rescue therapy during peroral endoscopic myotomy for achalasia patients with severe submucosal fibrosis

**DOI:** 10.1055/a-2239-4914

**Published:** 2024-02-02

**Authors:** Ahmad Madkour, Amr Elfouly, Hamdy Sayed, Ahmed El-tawansy, Ahmed Tawheed, Hassan Atalla

**Affiliations:** 1 575928 Endemic Medicine Department, Helwan University Faculty of Medicine, Cairo, Egypt; 2 575928 Anesthesia Department, Helwan University Faculty of Medicine, Cairo, Egypt; 3 68780 Hepatology and Gastroenterology Unit, Department of Internal Medicine, Mansoura University Faculty of Medicine, Mansoura, Egypt

Peroral endoscopic myotomy (POEM) is a well-established endoscopic therapy for achalasia with high success rates. Severe submucosal fibrosis correlates with prolonged procedure duration, higher perioperative complications, and even failed POEM. Methods to overcome severe submucosal fibrosis include changing to an anterior approach, simultaneous submucosal tunnel dissection 1 , open POEM 2 , and initial submucosal tunneling followed by through-muscle dissection 3 , with variable rates of success and complications. Here, we present a novel and successful method of initial intramuscular dissection (IIMD) in two patients with severe submucosal fibrosis where submucosal tunneling was not possible ( Video 1 ).

The initial intramuscular dissection technique is shown during peroral endoscopic myotomy for two longstanding achalasia patients with severe submucosal fibrosis.Video 1

Two patients with longstanding dysphagia were diagnosed with type I achalasia by high resolution manometry, with Eckardt scores of 9 and 12, respectively. Endoscopic evaluation revealed a dilated esophagus, with an inflamed unhealthy mucosa, and insufficient lifting after submucosal injection was encountered, with marked mucosal stiffness ( Fig. 1 ). Additional trials of distal submucosal injection close to the cardia also failed to give acceptable lifting. As a result, when mucosal incision was initiated, it failed to give sufficient space for the endoscope. Given the expected thick muscle layer, we decided to create an intramuscular tunnel at the entry point ( Fig. 2 ). The distal attachment cap was changed to a more tapered one, and partial muscle dissection at the apex of the mucosal incision was performed ( Fig. 3 **a,b** ) to create an intramuscular tunnel that allowed 4 cm of additional scope progression ( Fig. 3 **c,d** ) before it was possible to revert to the submucosal space ( Fig. 3 **e,f** ), with continuation for 3 cm on the gastric side. Full-thickness myotomy was carried out proximally and was continued for 2 cm on the gastric side.

**Fig. 1:**
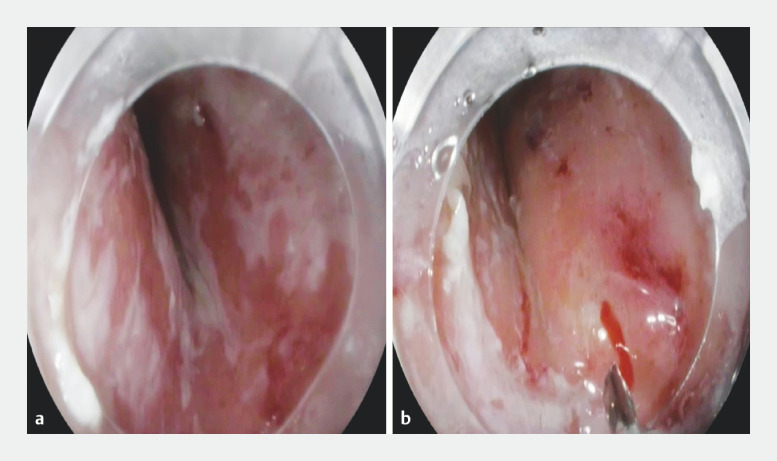
Endoscopic images showing: **a** the unhealthy stiff esophageal mucosa due to longstanding achalasia; **b** insufficient mucosal lifting, despite multiple trials of submucosal injection, because of severe submucosal fibrosis.

**Fig. 2:**
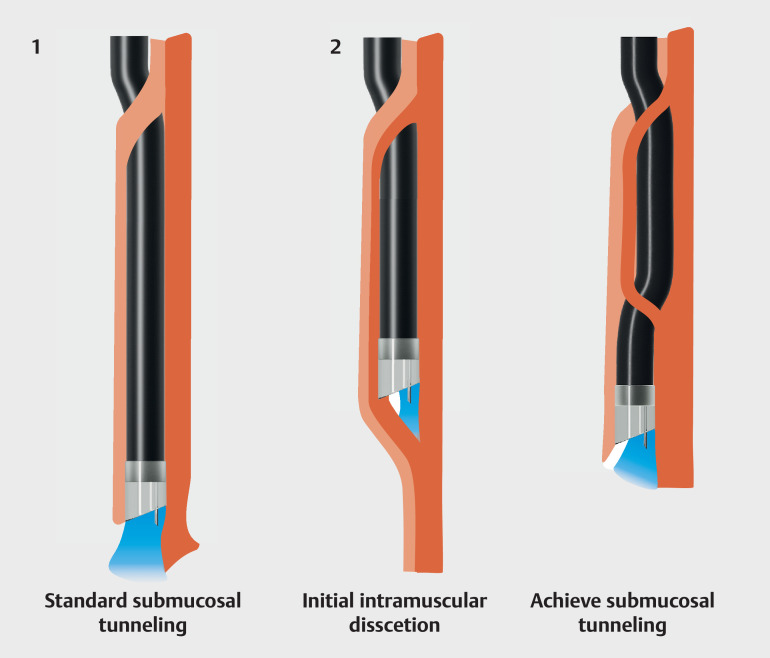
Schematic comparing the standard and novel initial intramuscular dissection techniques during peroral endoscopic myotomy showing: **a** standard submucosal tunneling; **b** the novel technique with initial intramuscular dissection followed by subsequent active submucosal tunneling.

**Fig. 3:**
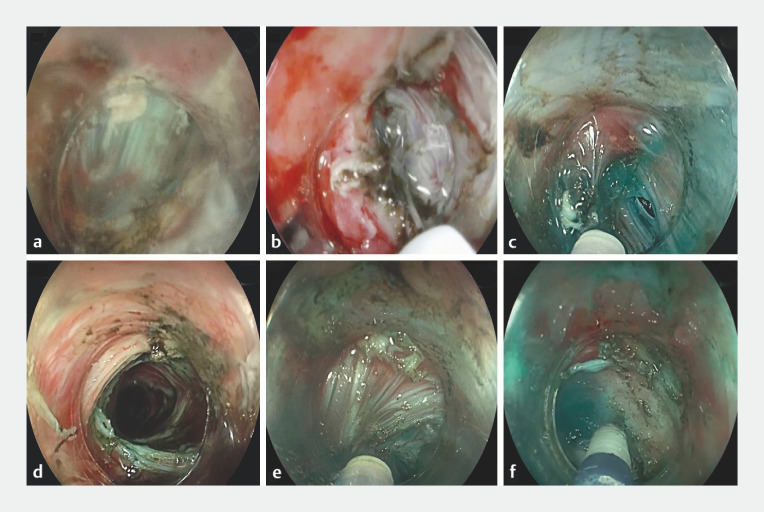
Endoscopic images showing: **a** the muscular layer in front of the scope immediately after the initiation of the mucosal incision giving no space for the scope; **b** initiation of intramuscular dissection at the apex of mucosal incision, with a more tapered attachment cap in position; **c** creation of the intramuscular tunnel, with progression toward the cardia; **d** the top of the already built intramuscular tunnel just below the mucosal incision; **e** a trial to divert the plane of dissection toward the superficial submucosal layer once possible; **f** successful navigation of the scope toward the submucosal layer, confirmed by methylene blue injection, which enabled further progression of the dissection plane toward the gastric side.

IIMD (“Madkour’s technique”) at the level of mucosal incision, first reported here to the best of our knowledge, may serve as a salvage option in cases where it is not possible to establish the submucosal tunnel owing to severe submucosal fibrosis in longstanding achalasia patients.

Endoscopy_UCTN_Code_TTT_1AO_2AN

## Notes

Endoscopy E-Videos https://eref.thieme.de/e-videos
*E-Videos* is an open access online section of the journal *Endoscopy* , reporting on interesting cases and new techniques in gastroenterological endoscopy. All papers include a high-quality video and are published with a Creative Commons CC-BY license. Endoscopy E-Videos qualify for HINARI discounts and waivers and eligibility is automatically checked during the submission process. We grant 100% waivers to articles whose corresponding authors are based in Group A countries and 50% waivers to those who are based in Group B countries as classified by Research4Life (see: https://www.research4life.org/access/eligibility/ ). This section has its own submission website at https://mc.manuscriptcentral.com/e-videos .
